# The use of augmented reality for limb and component alignment in total knee arthroplasty: systematic review of the literature and clinical pilot study

**DOI:** 10.1186/s40634-021-00374-7

**Published:** 2021-07-21

**Authors:** V. Iacono, L. Farinelli, S. Natali, G. Piovan, D. Screpis, A. Gigante, C. Zorzi

**Affiliations:** 1 Department of Orthopaedics IRCCS Ospedale Sacro Cuore Don Calabria, Negrar di Valpolicella, Italy; 2 Clinical Ortopaedics, Department of Clinical and Molecular Sciences, Università Politecnica Delle Marche, Ancona, Italy

**Keywords:** Augmented reality, Knee arthroplasty, Computer assisted surgery, Navigation knee arthroplasty

## Abstract

**Purpose:**

A systematic review of the literature has been carried out to assess the actual evidence of the use of augmented reality in total knee arthroplasty (TKA). We then conducted a pilot clinical study to examine the accuracy of the Knee + augmented reality navigation in performing TKA. The present augmented reality (AR) system allows the surgeon to view the tibial and femur axis superimposed on the surgical field through the smart glasses. It provides real-time information during surgery and intraoperative feedback.

**Methods:**

A systematic review of the PubMed, MEDLINE, and Embase databases up to May 2021 using the keywords “augmented reality”, “knee arthroplasty”, “computer assisted surgery”, “navigation knee arthroplasty” was performed by two independent reviewers. We performed five TKAs using the Knee + system. Patients were 4 females, with mean age of 76.4 years old (range 73–79) and mean Body Max Index (BMI) of 31.9 kg/m^2^ (range 27–35). The axial alignment of the limb and the orientation of the components were evaluated on standardized pre and postoperative full leg length weight-bearing radiographs, anteroposterior radiographs, and lateral radiographs of the knee. The time of tourniquet was recorded. The perception of motion sickness was assessed by Virtual Reality Sickness Questionnaire (VRSQ) subjected to surgeon immediately after surgery.

**Results:**

After duplicate removal, a total of 31 abstracts were found. However, only two studies concerned knee arthroplasty. Unfortunately, both were preclinical studies. Knee + system is able to perform a cutting error of less than 1° of difference about coronal alignment of femur and tibia and less than 2° about flexion/extension of femur and posterior tibial slope. The absolute differences between the values obtained during surgery and the measurement of varus femur, varus tibia, posterior slope, and femur flexion angle on post-operative radiographs were 0.6° ± 1.34°, 0.8° ± 0.84°, 0.8° ± 1.79°, and 0.4 mm ± 0.55 mm, respectively.

**Conclusions:**

On light of our preliminary results, the Knee + system is accurate and effective to perform TKA. The translation from pilot study to high-level prospective studies is warranted to assess accuracy and cost-effective analysis compared to conventional techniques.

**Level of evidence:**

IV

## Introduction

Limb and component alignment are fundamental factors for successful outcome and high survivorship of the implants in total knee arthroplasty (TKA) [1]. Over the last years, several techniques of computer-assisted surgical (CAS) navigation have been developed with the aim of improving accuracy and precision in component positioning [2]. Indeed, it has been reported a reduced overall rate of revision following TKA with the use of computer navigation [3]. However, most of CAS techniques required substantial cost, complex surgical setup with specialized training and increased operative time that limited their routine use. Among them, the technology of augmented reality (AR) is expanding, and its application in arthroplasty, especially in hip surgery, has gained increasing attention opening new opportunities in surgical planning and execution [4]. AR is defined as a technology, where the real world is augmented with virtual information by the use of smart glasses worn by the surgeon [5]. To the best of our knowledge, only one report has been reported about the use of AR in hip arthroplasty [4]. A systematic review of the literature has been carried out to assess the actual evidence of the use of augmented reality in knee arthroplasty. We then conducted a pilot clinical study on limited case series to analyze the accuracy of Knee + augmented reality system (Pixee medical company, Besancon, France) in limb and component alignment in TKA. We also evaluated intra operative perception of motion sickness of the surgeon and operative time.

## Material and Methods

### Systematic review

**Fig. 1:**
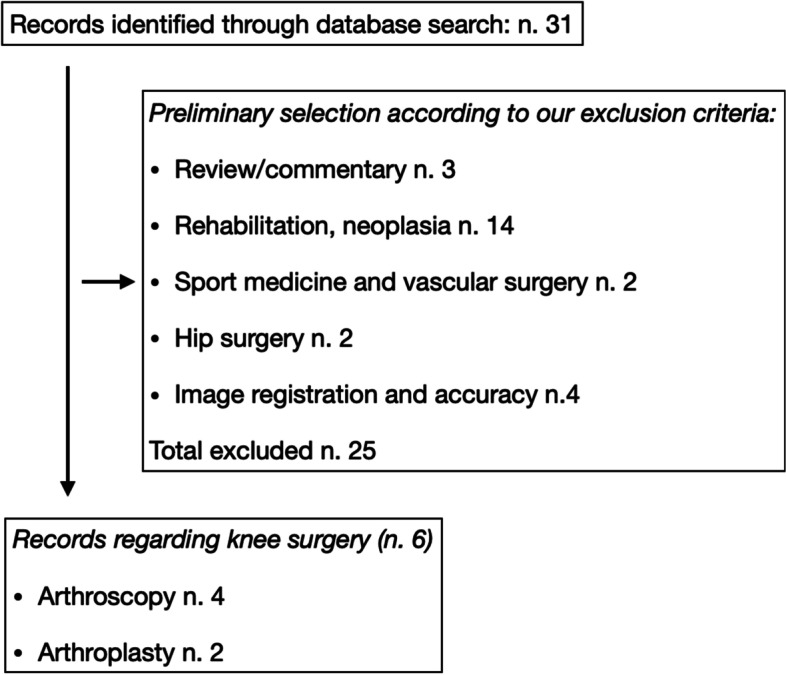
Selection protocol: abstract selection chart according to our inclusion criteria. Abstract inclusion and exclusion criteria are highlighted in the box

The review process was conducted according to the Preferred Reporting Items for Systematic Reviews and Meta-Analyses guidelines [6]. The Medline-PubMed, Embase, Web of Science and Cochrane Systematic Review databases were searched for studies published in English up to 31 May 2021. The primary search keywords were: “AUGMENTED REALITY” AND (“ORTHOPAEDIC” OR “ORTHOPAEDICS” OR “ORTHOPEDIC” OR “ORTHOPEDICS” OR “ARTHROPLASTY” OR “REPLACEMENT” OR KNEE”), “NAVIGATION KNEE ARTHROPLASTY” AND “AUGMENTED REALITY”. Papers were screened by title and abstract to identify relevant articles. Their reference lists were checked manually for additional articles. All the results were analysed independently by two revisors (LF and SN). A third revisor (CZ) have been consulted in case of incongruencies. Exclusion criteria were considered: clinical procedures not concerning knee arthroplasty (such as trauma or spine surgery). Inclusion criteria were: clinical studies conducted on humans or pre-clinical studies using augmented reality for knee arthroplasty. The selection process is described in detail in Fig. 1.

### Augmented reality navigation technique

The present study has been conducted using Knee + augmented reality navigation (Pixee medical company, Besancon, France). The Knee + system allows the surgeon to view the tibial and femur axis superimposed on the surgical field through smart glasses. It provides real-time information during surgery and intraoperative feedback. The surgeon could choose varus/valgus angle and posterior slope on tibial cut; the valgus distal femoral cut and flexion/extension of femoral component on femoral side.

**Fig. 2:**
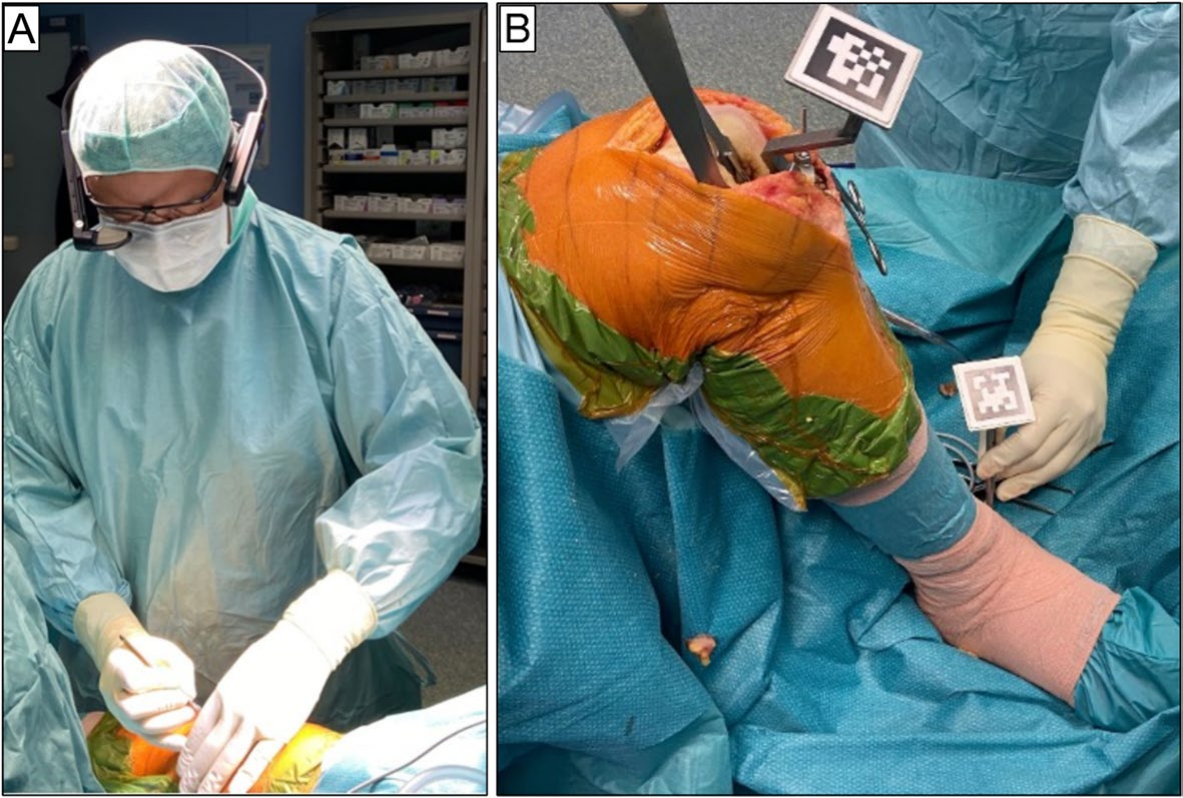
**A** Smart glasses worn by surgeon during surgery. **B** The surgeon registers bone landmarks consisted of lateral and medial malleolus using the pointer with QR-Code

The Knee + system consists of smart glasses worn (Fig. 2A) by the surgeon, a laptop and specific markers (QR-Code) that need to be connected with tibial and femur resection guide The Knee + system could be used in both femur or tibia-first TKA technique.

#### Tibia resection technique

**Fig. 3:**
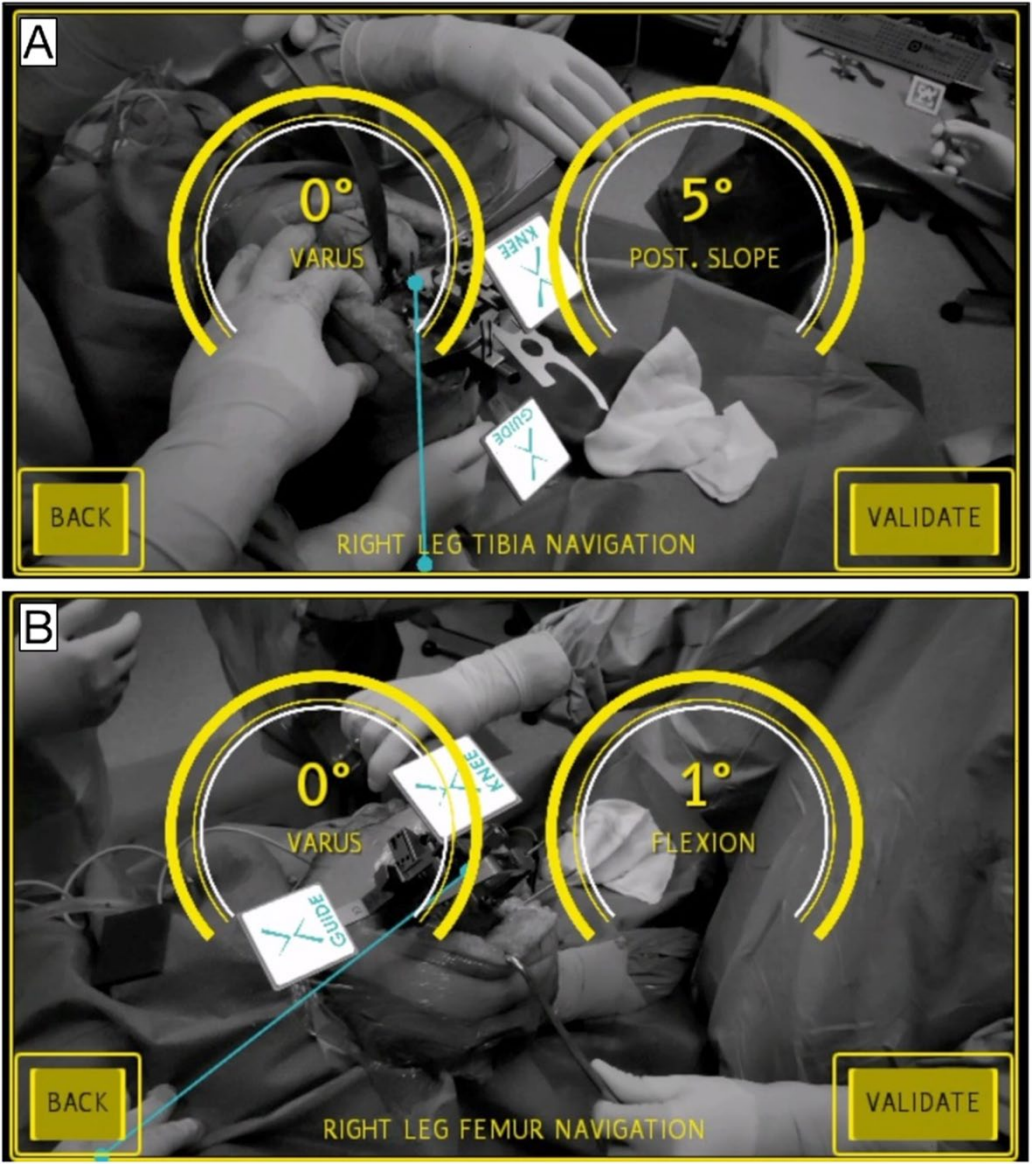
The Knee + system enables the surgeon to view the tibia (**A**) and femur (**B**) mechanical axis (blue line) superimposed on the tibia and femur on surgical field. In yellow circles, it has been indicated the coronal and sagittal alignment of cutting guide

**Fig. 4:**
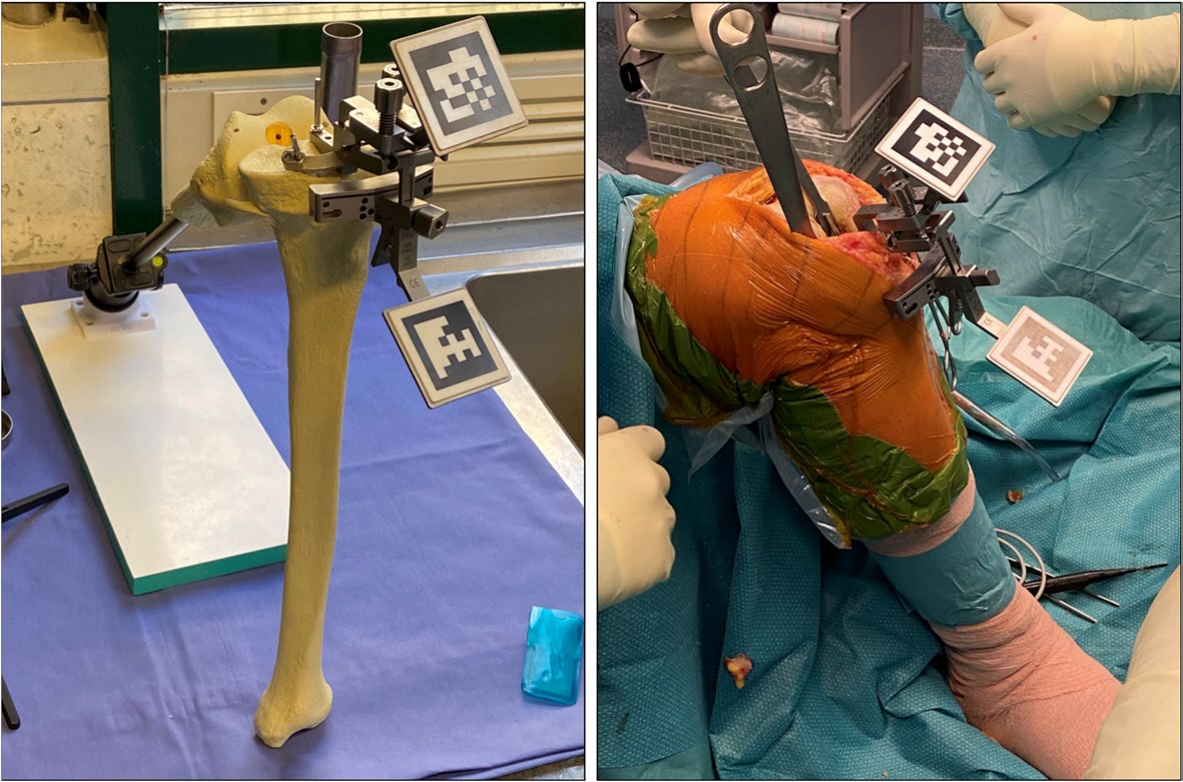
After registration phase, the surgeon could insert the tibial resection guide and fix the resection block when the desired angles of varus/valgus and tibial slope have been achieved

The surgeon inserts a tipped pin on tibial guide along the anatomic axes of the tibia. Subsequently, the surgeon registers bone landmarks consisted of lateral and medial malleolus using the pointer with QR-Code (Fig. 2B). It is important that surgeon sees both QR-codes on smart glasses during the registration phases. After completing registration, the Knee + system enables the surgeon to view the tibia mechanical axis superimposed on the tibia on surgical field (Fig. 3A). When the line does not fit the tibia properly, the surgeon can easily recognize that the registration is incorrect and could repeat it. By now, the surgeon could insert the tibial resection guide and fix the resection block when the desired angles of varus/valgus and tibial slope have been achieved (expected values) (Fig. 4.). After bone resection, the surgeon can check the varus/valgus and posterior slope using the marker (controlled values).

#### Distal Femur resection technique

The surgeon did insert a tipped pin in correspondence of the conventional femur entry point. A guide QR-code is inserted on surgical table. While keeping the pelvis stable, the surgeon should pivot the femur (circumduct hip) in an expanding spiral pattern until the registration is complete. After completing registration, the Knee + system enables the surgeon to view the mechanical axis of the femur superimposed on surgical field (Fig. 3B). By now, the surgeon could insert the femur resection guide and fix the resection block when the desired angles of distal femur cut, and flexion/extension have been achieved (expected values). After bone resection, the surgeon can confirm the angles achieved using the marker (controlled values). The absolute difference between expected and controlled values is defined as cutting error.

### Patients

From 5 April 2021 until 18 April 2021, we prospectively recruited 5 consecutive patients undergoing primary unilateral TKA with AR into this study. All patients were informed about the study and consented to participate. Patients were included irrespective of age, diagnosis, deformity and body mass index (BMI). We excluded revision surgery. All patients were operated on by the same orthopedic surgeon. In all patients an identical surgical technique and the Evolution® Medial-Pivot Knee Systems (MicroPort Orthopedics) TKA was used. The time of tourniquet was recorded. The perception of motion sickness was assessed by Virtual Reality Sickness Questionnaire (VRSQ) subjected to surgeon immediately after surgery [7].

The axial alignment of the limb and the orientation of the components were evaluated on standardized pre and postoperative full leg length weight-bearing radiographs, anteroposterior radiographs and lateral radiographs of the knee. In accordance to Bellemans et al. [8] the medial proximal tibial angle (MPTA), mechanical lateral distal femoral angle (mLDFA), joint line convergence angle (JLCA), anatomic-mechanical angle (AMA) and hip-knee angle (HKA) were determined based on the preoperative full-leg radiographs. Patients’ characteristics are summarized in Table 1. Postoperative HKA, alfa (α), beta (β), delta (δ) and gamma (γ) angle were determined based on the postoperative full-leg and knee radiographs. Accordingly, α is the medial angle between a line drawn parallel with the femoral component condyles and the anatomical axis of the femur. β is the medial angle between a line drawn parallel to the tibial component on the anterior–posterior radiograph and the anatomical axis of the tibia. Sagittal femoral γ is the proximal angle between a line drawn perpendicular to the distal cement interface of the femoral component and the femoral anatomical axis in the lateral radiograph. Sagittal tibial δ angle is the posterior angle between a line drawn parallel to the tibial component and the anatomical tibial axis in the lateral radiograph. In accordance to the Knee Society roentgenographic evaluation form, the coronal and sagittal femoral component alignments were rated as “aligned” if the α and γ angle were 90° ± 3°, respectively [9]. Patients were classified as outliers if the coronal and sagittal malalignments were greater than 3°. Conventionally, positive values of α and γ angle correspond to the valgus and flexion alignment of the femoral component, respectively.

**Table 1:** Patients characteristics

Patients	Age (y)	Gender	BMI (kg/m^2)^	HKA (°)	MPTA (°)	mLDFA (°)	AMA (°)	JLCA (°)
Patient 1	73	M	35.15	177	90	91	6	3
Patient 2	79	F	34.95	170	85	90	5	3
Patient 3	75	F	27.29	185	87	85	6	3
Patient 4	77	F	30.47	176	93	91	4	6
Patient 5	78	F	32	178	88	93	5	4

We calculated the absolute values of the differences between angles measured by AR and angles calculated on post-operative radiographs in terms of varus/valgus, flexion and posterior slope. The averages and standard deviations were calculated for each parameter.

## Results

### Systematic review

After duplicate removal, a total of 31 abstracts were found. Of these, 14 abstracts were excluded because they concerned rehabilitation, soft tissue tumors and not surgical technique or clinical results. 3 abstracts were excluded because they represented reviews without any clinical results. 2 abstracts were excluded because they concerned sport medicine, training and endovascular surgery. 2 abstracts were excluded because they related to hip arthroplasty. 4 abstracts were excluded because they concerned methods to improve accuracy of image registration. We found 6 abstracts regarding knee surgery. Specifically, 4 abstracts concerned knee arthroscopy and the effect of AR on minimally invasive knee surgery. Finally, only two studies concerned knee arthroplasty. Unfortunately, both were preclinical studies. The former reported by Fallavita et al. [10] only assessed the mechanical axes of lower limb on human cadaver limb. They found that AR was able to achieve a reliable mechanical axis deviation compared to computed tomography. However, they did not evaluate any femur or tibia resection. The latter reported by Tsukada et al. [11] reported a pilot study using sawbones where authors suggested that the AR may provide reliable accuracy for coronal, sagittal, and rotational alignment in tibial bone resection during total knee arthroplasty.

### Case series

Table 2 summarizes differences between the values obtained by AR system and the radiographic measurement values in terms of varus/valgus angle, flexion and posterior slope angle. In Table 3, we reported differences in absolute values as mean and standard deviation (SD) between expected and controlled values and between controlled and radiographic values. The time of tourniquet and VRSQ score have been reported in Table 4.

**Table 2:** Expected, controlled and radiographic measures obtained by Knee + system

	Expected values (°)				Controlled values (°)				Radiographic measures (°)			
Patients	Femur		Tibia		Femur		Tibia		Femur		Tibia	
	Varus	Flexion	Varus	Posterior slope	Varus	Flexion	Varus	Posterior slope	Varus	Flexion	Varus	Posterior slope
Patient 1	0	0	0	5	0	1	1	4	0	1	3	4
Patient 2	0	0	0	5	0	2	0	6	0	1	1	6
Patient 3	0	2	0	6	0	0	0	10	0	0	0	6
Patient 4	0	1	0	6	0	1	0	6	3	2	0	6
Patient 5	0	2	0	6	0	3	0	7	0	3	1	7

**Table 3:** Differences in absolute values as mean and standard deviation (SD) between expected and controlled values and between controlled and radiographic values

Variables	Expected vs Controlled values (°)	Controlled vs Radiographic values (°)
	Mean (SD)	Mean (SD)
**Varus femur**	0 (0)	0.6 (1.34)
**Flexion**	1.2 (0.83)	0.4 (0.55)
**Varus tibia**	0.2 (0.45)	0.8 (0.84)
**Tibia posterior slope**	1.4 (1.52)	0.8 (1.79)

**Table 4:** The time of tourniquet and VRSQ score

Patients	Time of tourniquet (min)	VRSQ score (range 0 -33)
Patient 1	70	12.5
Patient 2	65	4.17
Patient 3	50	6.67
Patient 4	45	7.5
Patient 5	48	4.17

## Discussion

The most important finding of the present study was that AR Knee + system could perform a cutting error of less than 1° of difference about coronal alignment of femur and tibia and less than 2° about flexion/extension of femur and posterior tibial slope. Moreover, all these measures are characterized by less than 1° of difference between controlled and radiographic values. All patients could be considered “aligned” in terms of α, β and γ. Our results were comparable to previous studies that assess the intra-operative cutting error of other conventional navigation systems in terms varus/valgus, flexion of femoral component and posterior tibial slope [12, 13]. Indeed, Hasegawa et al. [13] and Feichtinger et al. [12] reported an absolute differences in terms of cutting errors ranged from 0.5° to 1.2° in varus/valgus angle and from 0.7° to 1.4° in posterior slope using an image-less navigation system. Despite, AR is gaining popularity in orthopedic surgery, to the best of our knowledge, the present research is the first pilot study that assess the accuracy of AR system in performing knee arthroplasty.

Moreover, it needs to be highlighted that only two preclinical studies [10, 11] have been found from review process. From our results, we observed that the sagittal alignment planned is more difficult to achieve than coronal alignment. Hence, the cutting errors reported on flexion of femoral component and on posterior tibial slope were both greater than 1°. It has been well established that a considerable amount of error in sagittal alignment of femur and tibia could occur during bone cutting with the oscillating saw [14]. Indeed, Plaskos et al. reported that the movement of cutting blocks during cutting and the deflection of oscillating saw could determine a cutting error that might be 4° degrees [15].

The surgeon could visualize the virtual content superimposed on surgical field. Knee + system does not require neither preoperative computer tomography nor transcutaneous or trans-osseous pins in femur or tibia avoiding any related complications as pain or infection. In addition, the surgeon did not refer any vertigo or nausea at the end of surgery represented by low VRSQ scores; the time of tourniquet gets similar after the first two patients.

### Limitations

The present study had several limitations. We cannot assess the accuracy of Knee + augmented reality system because all surgeries were carried out by an experienced surgeon. It is known that the accuracy of bone resection depends on the experience of the surgeon [16]. Moreover, the present research consisted of a clinical pilot study characterized by a limited sample size. Analyzing the results of our review, it seems clear that the use of AR in knee arthroplasty is still in its infancy. This contributes to the difficulties in assessing its reliability and, more importantly, its accuracy.

From our clinical pilot study, the AR system in knee arthroplasty may become a useful alternative navigation system [11]. The translation from pilot study to high-level prospective studies is warranted to assess accuracy, limitations and cost-effective analysis compared to conventional techniques.
